# Current Role of Surgery in the Management of Oropharyngeal Cancer

**DOI:** 10.3389/fonc.2019.00388

**Published:** 2019-05-24

**Authors:** Wojciech Golusiński, Ewelina Golusińska-Kardach

**Affiliations:** Department of Head and Neck Surgery, Poznan University of Medical Sciences, Poznan, Poland

**Keywords:** oropharynx, cancer, surgery, TORS, TLM

## Abstract

In the last few decades, the surgical treatment of oropharyngeal squamous cell carcinoma (OPSCC) has undergone enormous changes. Until the 1990s, open surgery was the primary treatment for OPSCC. However, due to the potentially severe functional morbidity of this approach, open surgery was largely displaced by concurrent chemoradiotherapy (CRT) in the 1990s. At the same time, new, less-invasive surgical approaches such as transoral surgery with monopolar cautery began to emerge, with the potential to reduce functional morbidity and avoid the late-onset toxicity of CRT. More recently, the growing incidence of HPV-positive disease has altered the patient profile of OPSCC, as these patients tend to be younger and have a better long-term prognosis. Consequently, this has further bolstered interest in minimally-invasive techniques to de-intensify treatment to reduce long-term toxicity and treatment-related morbidity. In this context, there has been a renewed interest in the primary surgery, which allows for accurate pathologic staging and thus—potentially—de-intensification of postoperative CRT. The continuous advances in minimally-invasive surgical approaches, including transoral laser microsurgery (TLM) and transoral robotic surgery (TORS), have also altered the surgical landscape. These minimally-invasive approaches offer excellent functional outcomes, without the severe toxicity associated with intensive CRT, thus substantially reducing treatment-related morbidity. In short, given the increasing prevalence of HPV-positive OPSCC, together with the severe long-term sequela of aggressive CRT, surgery appears to be recapturing its previous role as the primary treatment modality for this disease. While a growing body of evidence suggests that TLM and TORS offer oncologic outcomes that are comparable to CRT and open surgery, many questions remain due to the lack of prospective data. In the present review, we explore the emerging range of surgical options and discuss future directions in the treatment of OPSCC, including the most relevant clinical trials currently underway.

## Introduction

Oropharyngeal squamous cell carcinoma (OPSCC) has traditionally been treated with open surgery due to the limited access to this complex anatomic location. However, the invasiveness of open techniques can cause severe functional morbidity, most notably functional compromise of speech and swallowing, with high complication rates (1). Moreover, open surgery also requires complex reconstruction of the resection defect. In this context, clinical trials conducted in the late 1990s demonstrated that concurrent chemoradiotherapy (CRT) achieved locoregional control and survival outcomes that were comparable to open surgery but with less morbidity (2). As a result, CRT began to gradually replace open surgical techniques. However, despite the initial promise, over time it became clear that CRT was associated with severe late onset toxicity, particularly acute mucositis and severe dysphagia, with a major negative impact on patient quality of life (QoL) (3–6).

The incidence of OPSCC has risen in recent decades due to the increase in human papillomavirus (HPV)-positive disease, which now accounts for 70% of newly-diagnosed cases (1). The growing incidence of HPV-positive OPSCC, which has doubled in the last decade, has radically altered the prognosis and treatment of this disease (7). Historically, most cases of OPSCC were associated with tobacco and alcohol use, which predominantly affected older patients. By contrast, nowadays most patients diagnosed with tonsillar or base of tongue disease—the most common tumor sites in OPSCC—are HPV-positive. Patients with HPV-positive disease are usually diagnosed at earlier stages and tend to be younger, more highly-educated, and more likely to be non-smokers; more importantly, they have a markedly better prognosis than HPV-negative patients (8–10). Given the younger age and better long-term survival of these patients, treatment-related toxicity is a highly relevant consideration in treatment selection. In this regard, although HPV-positive tumors are more susceptible to the effects of radiation (8), the risk of the late onset adverse effects of CRT, which include osteoradionecrosis, fibrosis, trismus, xerostomia, and dysphagia, must be considered carefully when selecting the treatment approach (11).

The emergence of this new patient profile has given rise to a search to de-intensify treatment to reduce long-term toxicity and improve QoL, with numerous de-intensification trials currently underway (12). In this context, there has been a renewed interest in the surgical management of this disease, in part because primary surgery allows for accurate pathologic staging, which—depending on the pathologic findings—may allow for de-intensification of postoperative chemotherapy and/or radiotherapy. Similarly, there is a growing interest in minimally-invasive surgical approaches, such as transoral laser microsurgery (TLM) and transoral robotic surgery (TORS), which are associated with decreased treatment-related morbidity (13, 14).

In the present review we describe the range of surgical options currently available to treat OPSCC, with a focus on minimally-invasive techniques. In addition, we discuss future directions in the treatment of OPSCC, including the most relevant clinical trials currently underway.

## Surgical Options for the Management of Oropharyngeal Cancers

One the main advantages of surgery over primary CRT is that it allows for pathological staging, thereby providing an accurate assessment of the extent of disease, which may permit treatment de-intensification in some patients, an important benefit given that most of the long-term functional impairment and poor QoL in patients treated for OPSCC are treatment-related (15). Indeed, the potential to de-escalate adjuvant therapy is a major reason to consider primary surgery rather than primary CRT.

**Figure 1:**
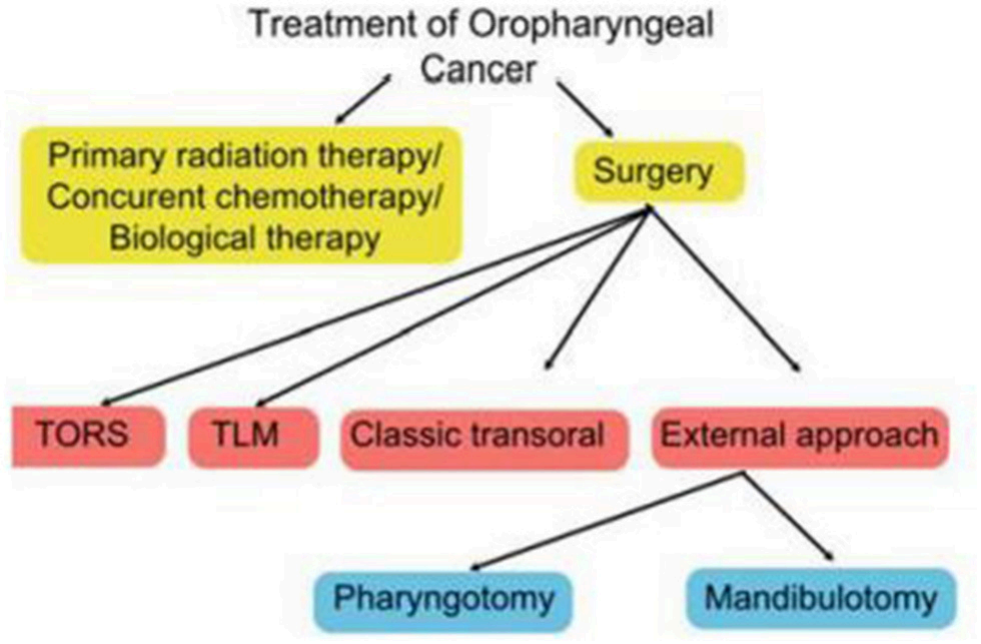
Scheme of treatment of oropharyngeal cancer.

The specific surgical technique will depend on a wide range of factors, including disease stage, anatomic location, patient characteristics (age, occupation, general health, presence of co-morbid conditions), HPV status, and patient and clinician preferences (16). The expertise and equipment available at the treating centre is also an important consideration. Figure 1 shows the main treatment options.

Surgery for OPSCC can be broadly divided into open or transoral surgical techniques. Open surgery generally involves mandibulotomy or pharyngotomy while a range of transoral techniques are available, including classic transoral surgery with monopolar cautery, TLM, and TORS. Although open surgery is primarily—but not solely—used to treat advanced cancers (stage III or IV) or for salvage therapy, several studies have shown that some early-stage primary lesions may be amenable to surgical extirpation (17, 18). By contrast, in some advanced cases, non-surgical treatment may be more appropriate if the likelihood of achieving a cure is small.

### Selection of the Surgical Technique

Given the complex anatomy and functional importance of the oropharynx, a wide range of surgical techniques are available to manage OPSCC. Open surgery, which includes mandibulotomy, mandibulectomy, and/or pharyngotomy, may be performed to treat advanced tumors or for surgical salvage after failed radiotherapy or failed CRT. However, due to the potential for significant treatment-related morbidity—including prolonged hospitalization, cosmetic deformity, gastrostomy tube and tracheostomy dependence—there is a growing preference for transoral approaches to decrease treatment-related morbidity. The advantages of transoral surgical techniques vs. open approaches include decreased damage to the musculature and to the major neurovascular structures and normal tissues, as well as faster recovery and shorter hospital stays (19, 20). Indeed the emergence of TLM—and more recently, TORS—has reduced the role of open surgery as initial therapy for OPSCC. Although minimally-invasive surgical approaches are currently limited to early-stage disease contained within the oropharynx, some authors have described the use of these techniques in well-selected patients with advanced tumors (21, 22).

### Transoral Laser Microsurgery

Classic transoral surgery, which is performed using traditional instrumentation and monopolar cautery, was first developed in an effort to minimize the morbidity associated with open surgery. Although this technique has important limitations, primarily poor visualization, and limited maneuverability, it is still in use today mainly limited to tumors that can be visualized directly and manipulated with standard instrumentation and lighting (23, 24).

The drawbacks of the classic transoral approach led to the development of TLM in the late 1990s, in which advanced retractors provide surgical access and visualization. TLM offers improved visualization and laser resection, which is more precise than monopolar cautery (2). The comparative advantages of TLM include a greatly reduced risk of fistula, abscess, and osteoradionecrosis, as well as a shorter length of stay in the hospital, which can substantially reduce treatment-related expenses (25). The most important drawbacks of TLM are related to difficulties in achieving hemostasis (which may require diathermy or surgical clips) and in tissue manipulation (26). In addition, in the past, the long, rigid equipment and narrow field view of older laryngoscopes made it difficult to maneuver within the complex anatomy of the oropharynx. However, the development of newer retractors and more advanced laryngoscopes has greatly improved access and maneuverability (27). Notwithstanding these limitations, TLM remains widely used, particularly in Europe (28).

### Transoral Robotic Surgery

TORS was first described by Hockstein (29) in 2005 in a canine model, with the first treatment in a human described in the same year (30). TORS was first approved by the United States Food and Drug Administration (FDA) in 2009 for the treatment of T1-T2 tumors of the oropharynx.

Transoral approaches have several important advantages over open surgical techniques, primarily significantly less cosmetic and functional morbidity. Similarly, TORS has several key advantages over TLM, including: (1) 3D panoramic vision, (2) improved range of motion (360° robotic arm movement) due to the lack of supination and limited pronation, (3) better optics, and (4) hand tremor filtration, and (5) easier en bloc resection (31). Although the body of evidence to support TORS is growing, especially for early-stage tumors, it is important to stress the need for more data on long-term oncological and functional outcomes. Table 1 summarizes the main studies conducted to date (14, 32–38)—two of which were prospective (34, 37, 38)— that have reported survival outcomes for TORS.

**Table 1:** Oncological outcomes of TORS for oropharyngeal squamous cell carcinoma.

**Study**	**Year**	**Study design**	**Treatment technique**	**Treatment subsite**	**Tumor stage**	***N***	**Follow-up**	**Local control**	**Locoregional control**	**Survival**
Weinstein (38)	2012	Prospective	TORS	Tonsil BOT Glossotonsillar sulcus Palate	T1-T4	30	2.7 years	97%	97%	OS = 100%
de Almeida (14)	2015	Retrospective, multicentre	TORS	Oropharynx Tonsil BOT	Tx–T4 (≤T2 = 86.2%	364	20 months	95.6% (3 year)	3-years: 88.8%	OS (3 year) = 87.1%
Kass (21)	2016	Retrospective	TORS = 42% TLM/transoral = 45% Open = 13%	Oropharynx	T1-T2	143	41 months	NR	NR	RFS (3 year) = 87% (TORS)
Dabas (34)	2017	Prospective	TORS	BOT	T1-T2	57	29 months	NR	95.8%	DFS = 89.6% OS = 93.8%
Moore (35)	2017	Retrospective	TORS	Tonsil BOT	Tx-T4	314	3.3 years	NR	92% (5 years)	OS = 86% DFS = 94%
Min (37)	2017	Prospective	TORS	Pyriform sinus Pharyngeal wall	T1-T4	38	60 months	97.4%	NR	OS = 55.3% DFS: = Stage I-II/III-IV: 100%/68.6%
Doazan (36)	2018	Retrospective, multicentre	TORS	Supraglottic	T1-T3	122	42.8 months	2 year: 94.3% 5 year: 90.2%	2 year: 91.8% 5 year: 93.5%	2/5 year OS = 86.9%/78.7% 2/5 year DFS = 95.1%/94.3%
Baliga (32)	2018	Retrospective (cancer registry)	TORS	Oropharynx	T1-T2	2680	31.4 months	NR	NR	5 year OS = 84%

*NR, not reported; TORS, transoral robotic surgery; DFS, disease-free survival; RFS, relapse-free survival; OS, overall survival; BOT, base of tongue*.

Despite the growing popularity of TORS, the bulk of the evidence to support this technique is for early-stage OPSCC (39). Moreover, most patients still require postoperative radiotherapy (PORT), although potentially with lower doses than used in conventional CRT. TORS—like all surgical procedures—poses a risk of severe adverse effects, most commonly postoperative hemorrhage, a potentially fatal complication whose reported incidence rate ranges from 3 to 8% (40–43). Other potential complications of TORS include aspiration pneumonia, dysphagia, pharyngeal fistula, temporary tracheostomy, and the need for conversion to open surgery (44, 45). TORS has other drawbacks, not least of which is the expense of the robotic equipment, the bulky equipment, and the use of cauterization rather than laser (46). Cautery requires wider and deeper incisions than laser, causing more thermal damage to the surrounding tissues. Although a few reports have described the use of CO_2_ laser with TORS, this technique is not widely available and remains experimental (47, 48).

### Patient Selection for Minimally-Invasive Approaches

The principle underlying all minimally-invasive approaches is to achieve maximum exposure while minimizing surgical morbidity. In this sense, a major consideration in patient selection is whether the surgery is likely to reduce or eliminate the need for adjuvant CRT. The key consideration for all endoscopic techniques is tumor access (28), which must permit en bloc resection of the primary tumor with sufficient margins (≥ 5 mm in all planes) without a high risk of causing long-term functional impairment. If these conditions cannot be met, then other treatment modalities, including open techniques or CRT, must be considered.

Surgical feasibility is generally determined by a comprehensive physical examination combined with endoscopy and imaging. The tumor and adjacent critical structures should be visible through the robotic endoscope and accessible for resection using the robotic instruments. Finally, given that the most serious complications of TORS is bleeding, access must be sufficient to ensure hemostasis.

Rich et al. (49) identified eight factors (known as the 8 Ts: teeth, trismus, transverse dimensions [mandibular], tori, tongue, tilt, treatment [prior radiation], and tumor) that should be considered to ensure proper endoscopic access in patients undergoing TLM. These criteria are also generally applicable to TORS. Factors that hinder access to the oral cavity (e.g., trismus) or the ability to achieve adequate patient positioning (e.g., limited neck extension) are important contraindications. Other pre-operative exclusion criteria for TORS include morbid obesity (body mass index > 40), micrognathia, microstomia, and craniofacial abnormalities, all of which may prevent robotic access (2, 41, 50). Mandibular body height, hyoid-mental length, and neck circumference may also effect eligibility (51).

Tumor characteristics also play a key role in determining suitability for TORS. For this reason, it is essential to assess the potential involvement of neighboring anatomical structures. Weinstein and colleagues (52) classified the contraindications for TORS into three categories: vascular localization, functional limitations, and oncologic contraindications (Table 2). As those authors observed, even in patients with ostensibly resectable tumors, functional outcomes and surgical morbidity should both be carefully considered when planning treatment.

**Table 2:** Contraindications for TORS.

**VASCULAR**
•Tonsillar involvement with a retropharyngeal carotid artery
•Tumor located at the midline of the tongue base or vallecula
•Tumor located adjacent to the carotid bulb or internal carotid artery
•Carotid artery enveloped by tumor or metastatic lymph nodes
**FUNCTIONAL**
•Tumor resection requiring ≥50% of the deep tongue base musculature or posterior pharyngeal wall
•Resection of the tongue base and entire epiglottis
**ONCOLOGICAL**
•Stage T4b
•Unresectable neck disease
•Multiple distant metastases
•Neoplastic-related trismus
•Prevertebral fascia involvement
•Mandible or hyoid involvement
•Tumor extension to lateral neck soft tissues
•Involvement of the eustachian tube

## Early-Stage OPSCC

Until very recently, definitive radiotherapy or CRT were considered the main treatments for early-stage (T1-2 N0-1) OPSCC (23). However, the long-term toxicity of these modalities—together with the important consequences of failed radiotherapy for subsequent salvage surgery (6)—have generated more interest in minimally-invasive surgical approaches, which can provide more accurate pathologic staging, potentially eliminating the need for PORT or CRT or at least allowing for lower dose therapies. Accordingly, current recommendations from the US National Comprehensive Cancer Network (NCCN) guidelines for early-stage OPSCC (53) are either definitive radiotherapy or primary surgery (transoral or open, with or without ipsilateral or bilateral neck dissection). Adjuvant CRT is recommended only in cases in which extracapsular dissemination or positive margins unsuitable for re-excision are detected during surgery. Although the NCCN guidelines recommend either transoral or open surgery, the transoral approach with elective neck dissection is generally considered the surgical treatment of choice in suitable patients with early-stage OPSCC.

Retrospective data on locoregional control and survival rates in early-stage OPSCC have shown equivalent efficacy between radiotherapy and surgery, although no prospective randomized controlled trials are available yet to confirm these results (54). Early-stage disease should ideally be treated with single modality therapy, either primary surgery or radiotherapy. Although there is a notable lack of high-quality comparative studies, retrospective data indicate equivalent 5-year DSS rates, ranging from 81 to 100% for primary surgery (with adjuvant therapy when necessary) and 77–89% for primary radiotherapy (55). Morisod et al. (56) conducted a meta-analysis of 12 retrospective studies to compare radiotherapy to transoral surgery (TLM and classic transoral approaches). Of those 12 studies, seven evaluated radiotherapy (*n* = 729 patients) and five transoral surgery (*n* = 276). The 5-year disease-specific survival (DSS) rate was 90.4% (95% confidence interval [CI], 85.6 to 95.2%) in the radiotherapy group vs. 89.6% (95% CI, 81.8 to 97.3%) in the transoral surgery group (*p* > 0.05). The authors concluded that the available data suggest an equivalent efficacy between surgery and radiotherapy in terms of disease control for early-stage OPSCC. However, it should be noted that this meta-analysis did not include TORS and, in general, the level of evidence (grade 4) was low. A retrospective review conducted by De Almeida et al. (14) of 410 patients who underwent TORS reported 3-year overall survival (OS) and DSS rates of 87.1 and 94.5%, respectively, which are equivalent to or better than those achieved with definitive radiation (1). The largest study of TORS in OPSCC conducted to date (*n* = 2680) was performed by Baliga et al. (32) who reviewed the National Cancer Database registry to identify patients diagnosed and treated for clinical T1-T2, N0-N2b OPSCC between 2010 and 2014. Most patients (14,470; 84.4%) received primary radiotherapy while 2,680 (15.6%) underwent TORS. At a median follow-up of 31.4 months, propensity score matching showed that both techniques yielded similar 5-year OS (81% for radiotherapy vs. 84% for TORS; log rank, *p* = 0.10). The early results from that study suggest that TORS may induce less long-term functional impairment than radiotherapy, an important consideration given the comparable oncologic results (55).

In view of the evidence described above, the preferred surgical approach for early stage tumors is either TLM or TORS, which have been shown to achieve results that are comparable to both open surgery and primary radiotherapy (54). Given that up to 30% of patients with cT1–T2 N0 disease will present occult nodal disease, ipsilateral selective neck dissection is recommended in those who are treated with primary surgery. Contralateral neck dissection may also be considered in midline tumors for pathological staging.

## Advanced-Stage OPSCC

Surgical treatment options for advance OPSCC (stage III and IV) include surgery plus PORT, with or without chemotherapy. Both CRT and conventional surgery can be used in advanced cancers (T3-4a, N0-N1), depending on the tumor localization and on the specific expertise of the treating hospital (57, 58).

Surgery requires an extensive resection of the visible or palpable tumor. A 1.5–2 cm macroscopic free surgical margin should be applied, if feasible, with frozen section analysis to assess the surgical margins. In most advanced stage tumors, an open approach with lip-splitting mandibulotomy is necessary to achieve adequate visualization. This means that is Mandibulotomy is the treatment of choice in advanced base of tongue or tonsillar complex tumors; however, lateral pharyngotomy may also be used for the tonsillar complex. Partial mandibular resection may be necessary if bone infiltration is detected.

Depending on the tumor location, either transoral or open surgery, generally followed by PORT or CRT, can be used. Several studies have reported promising results for transoral resection—TORS or TLM—of base of tongue, tonsil and pharyngeal wall primary tumors (21, 59). The indication for advanced stage soft palate tumors is generally transoral resection with radical neck dissection. In patients who are not eligible for transoral resection, such as those with large primaries, CRT should be considered. Advanced base of tongue tumors are usually managed with an open surgical approach (i.e., anterior mandibulotomy) with radical neck dissection and free flap reconstruction. Advanced tonsillar tumors may also be treated with transoral resection with radical neck dissection followed by reconstruction, and radial forearm free flap (RFFF) but some require anterior mandibulotomy with radical neck dissection and reconstruction with RFFF.

Evidence suggests that more than 50% of patients with advanced-stage OPSCC will develop nodal metastasis (60). For this reason, patients with nodal involvement should undergo a modified neck dissection or, at a minimum, selective dissection of levels I–IV. The surgical management of N0 neck disease is elective, generally involving selective dissection of levels II, III and IV (57).

While some authors have reported the use of TORS in advanced OPSCC, most of these studies involved patients with low T stage but advanced cervical disease (58, 61, 62). In these studies, TORS was used as the first-line therapy, followed by adjuvant radiation and chemotherapy when necessary. Hutechson et al. conducted a systematic review of functional outcomes after TORS for OPSCC in which they concluded that, despite the promising results to date, randomized, baseline-adjusted outcomes are needed to determine functional differences among patients treated with primary TORS vs. non-surgical organ-preserving approaches (63).

## Surgical Salvage

Despite the changing role of surgery in the last two decades, it remains the backbone of salvage therapy for recurrent OPSCC. Although the emergence of organ preservation strategies has decreased the role of primary surgery in advanced OPSCC, up to 60% of patients with advanced-stage tumors still develop recurrent disease and thus surgery is generally considered the treatment of choice for resectable tumors (64). However, because the vast majority of patients with OPSCC receive radiation therapy (either primary or adjuvant) at some point in their treatment, the sequelae associated with this treatment pose many challenges for successful salvage surgery, including tissue edema, necrosis, and chondritis. Patients treated with prior radiotherapy have more postoperative complications after salvage surgery and poorer wound healing (64).

Due to the need for radical resection, open surgery has long been the treatment of choice for salvage therapy. In recurrent OPSCC, it is essential to select a surgical approach that provides adequate access and visualization of the tumor. The available options include open techniques via a transcervical, lingual release approach or, in some cases, mandibulotomy and/or pharyngotomy with free flap reconstruction and tracheostomy, all of which imply a high risk of complications, as evidenced by the high complication rates (around 50%), with poor survival outcomes (5-year DFS rates of 20%) (1).

TORS and TLM may provide a viable alternative to open surgery in the salvage setting, but this indication is not yet well-established due to the limited data. The transoral route is generally limited to patients with stage rT1 or rT2 disease, whereas more aggressive surgical approaches (transcervical, with or without mandibulotomy) are more appropriate in patients with more advanced stages (rT3 and rT4) (65). However, when feasible, TORS may achieve acceptable oncologic outcomes and better functional outcomes than traditional open surgical approaches (27), and thus it seems reasonable to prefer transoral to open approaches in well-selected patients to reduce treatment-related complications (particularly fistulas) (66). Recently published multidisciplinary guidelines in the United Kingdom (65) suggest that—in carefully selected patients—transoral surgery appears to be an effective alternative to open surgery for the management of recurrent OPSCC.

The evidence base to support TORS in the salvage setting is still limited but expanding. White et al. carried out a retrospective, multi-institutional case-control study (67) to compare oncologic and functional outcomes in 128 patients with recurrent OPSCC treated with TORS (*n* = 64) or open surgery (*n* = 64). The TORS group had a significantly lower (*p* < 0.001) incidence of tracheostomy and feeding tube use, shorter overall hospital stays (3.8 vs. 8.0 days), decreased operative time (111 vs. 350 min), and less blood loss (49 vs. 331 mL). In addition, the 2-year DFS was significantly higher in the TORS group (74 vs. 43%; *p* = 0.01). Although these differences appear to favor TORS, the authors emphasize that selection bias (patients with less well-defined tumors underwent open surgery) likely had a significant impact on the difference between the two groups in terms of DFS and in the better functional outcomes obtained in the TORS group.

Meulemans et al. (68) evaluated functional and oncologic outcomes of 86 patients who underwent primary and salvage TORS at three institutions in Belgium. In the 30 patients who underwent salvage TORS most of the tumors were stage cT1-rT1 (60.0%)/pT1-rpT1 (60.0%) or cT2-rT2 (40.0%)/pT2-rpT2 (23.3%) and cN0 (83.3%). Most (63.3%) of these patients did not receive any adjuvant therapy. At a median follow-up of 21.2 months, functional results were excellent (no cases of definitive tracheostomy, long-term tube feeding in 20% of cases). Estimated 2-year OS (standard error [SE]), was 73.5% (10.9%), 2-year DSS was 93.3% (6.4%), and 2-year DFS was 75.8% (9.7%).

More recently, Paleri et al. (69) evaluated 26 patients considered potential candidates for TORS in salvage OPSCC. Of these, 21 underwent TORS and 5 open resection (4 due to unsuitable anatomy or tumor extension). At 42.6 months of follow-up, OS was 48.2%, with local control and DSS rates of 76.6 and 77.1%, respectively (69). Based on those findings, the authors concluded that TORS is a valid management option for residual and recurrent OPSCC. They also noted that oncologic outcomes were comparable to open surgery and TLM, with the added advantage of en bloc resection, the ability to perform intraoperative ultrasound imaging, and to inset free flaps without mandibular split.

In the salvage setting, the early evidence for TORS in terms of perioperative and functional outcomes is highly promising; however, the available data are too preliminary to make any definitive conclusions and selection bias can have an important impact. Long-term oncologic outcomes from larger studies are needed to better establish the benefits of TORS in this setting and to more clearly define selection criteria for TORS vs. open surgery (65).

Interestingly, a recent meta-analysis conducted by Ibrahim and colleagues (70) to compare TLM, TORS, and conventional direct transoral oropharyngectomy for salvage treatment found that none of these techniques were significantly superior to the others in terms of functional outcomes. As a result, those authors concluded that surgeon experience, resource availability, and patient preferences are the main factors that determine the choice of transoral technique.

## Future Directions

### Current Clinical Trials

HPV-positive disease is more sensitive to chemotherapy and radiotherapy than HPV-negative disease and thus these patients have a much better prognosis with conventional treatments and could be good candidates for treatment de-intensification. However, the results of two recently published clinical trials (71, 72) showed that substituting cetuximab for cisplatin to reduce toxicity resulted in significantly worse survival outcomes, without any differences in acute or late toxicity. Nevertheless, several other treatment de-intensification strategies, including the use of minimally-invasive surgery followed by reduced doses of adjuvant therapy (chemotherapy and/or radiotherapy) are currently being investigated in several ongoing clinical trials designed to determine whether it is possible to de-escalate adjuvant treatment based on the pathologic findings of surgery. A search on clinicaltrials.gov (as of October 10, 2018) a total of 30 studies either underway or completed. Among the most relevant of these trials are the following:

The ADEPT (Adjuvant De-escalation, Extracapsular Spread, p16 Positive, Transoral) trial (**NCT01687413)**, a phase III trial which is examining HPV-positive, high-risk OPSCC patients treated with transoral surgery with negative margins. The main aim is to determine if it is possible to omit postoperative chemotherapy.

The ECOG 3311 is a phase II trial **(NCT01898494)** involving 511 patients with stage III/IV HPV-positive OPSCC treated by transoral surgery and neck dissection. The aim of this de-intensification trial is to determine if similar outcomes can be achieved with a lower dose of postoperative radiotherapy.

The Oropharynx: Radiotherapy vs. Trans-Oral Robotic Surgery (ORATOR) trial **(NCT01590355)** is a single-institution trial comparing QoL and survival outcomes in OPSCC treated with transoral surgery (TORS) or primary radiotherapy.

The PATHOS (Post-operative Adjuvant Treatment for HPV-positive Tumors) trial **(NCT02215265)** involves patients with HPV-positive cancer (T1-3, N0-2b) treated by transoral surgery and neck dissection. The aim of that trial is to identify patients in whom adjuvant treatment can be de-intensified after transoral surgery. Patients are stratified into 3 groups according to their pathologic results to receive either 50 or 60 Gy of adjuvant radiotherapy, with or without chemotherapy.

Transoral head and neck surgery compared with radiotherapy—EORTC “The best of” trial **(NCT02984410)**. The primary endpoint of this trial is swallowing function, and the secondary endpoint is to compare overall survival between the two treatment methods.

## Conclusions

In the context of the rising incidence HPV-positive oropharyngeal cancer, together with the potentially severe long-term sequela of aggressive CRT and the rapid improvement in minimally-invasive robotic techniques, there has been a renewed interest in surgical approaches to treating OPSCC. An important advantage of upfront surgery over CRT is the potential to stratify patients by pathological risk, which may allow for treatment de-escalation to reduce long-term morbidity.

Clearly, long-term comparative data on the functional and oncological outcomes of emerging treatment modalities for advanced-stage OPSCC—primarily minimally-invasive techniques—are needed. When these become available, it will facilitate treatment selection aimed at achieving the best clinical outcomes with the least treatment-related morbidity.

## Author Contributions

WG: the concept of the paper, substantive content. EG-K: literature review, figures, and table.

### Conflict of Interest Statement

The authors declare that the research was conducted in the absence of any commercial or financial relationships that could be construed as a potential conflict of interest.
